# Utilizing Maternal Morbidity as a Novel Screening (MMS) Tool for Predicting Peripartum Morbidity at a Rural Tertiary Care Teaching Hospital in Central India

**DOI:** 10.7759/cureus.65887

**Published:** 2024-07-31

**Authors:** Arti M Wasnik, Neema Acharya, Manjusha G Mahakalkar

**Affiliations:** 1 Obstetrics and Gynaecology, Kasturba Nursing College, Sevagram, Wardha, IND; 2 Obstetrics and Gynaecology, Jawaharlal Nehru Medical College, Datta Meghe Institute of Higher Education and Research, Wardha, IND; 3 Obstetrics and Gynaecological Nursing, Smt. Radhikabai Meghe Memorial College of Nursing, Datta Meghe Institute of Higher Education and Research, Wardha, IND

**Keywords:** non-triggered group, triggered group, peripartum morbidity, maternal morbidity tool, meows chart

## Abstract

Background

The majority of complications and deaths related to childbirth are concentrated in developing and disadvantaged nations, where the rates are unacceptably elevated. These incidents predominantly occur in the vicinity during the intrapartum period and immediately after childbirth. The peripartum period is especially critical for expectant mothers, as it represents the time when a significant number of complications and deaths occur. This study aimed to develop, validate, and assess the efficacy of the maternal morbidity screening (MMS) tool for predicting peripartum morbidity.

Methodology

The study was conducted in two phases: Phase one involved developing, validating, and piloting the MMS tool, while Phase two focused on evaluating and comparing the MMS tool with the modified early obstetric warning system (MEOWS) chart for predicting peripartum morbidity. An observational analytical clinical study design was utilized.

Result

In Phase one, the MMS tool was developed and validated by subject experts, resulting in a reliability score of 0.90. Therefore, the tool was deemed reliable and valid. Phase two results revealed that obstetric morbidity in the maternal morbidity group was 66.66%, higher than the 32% observed with the MEOWS chart. The MMS tool demonstrated significantly higher sensitivity at 95.24%, specificity at 89.50%, and predictive value at 98.50%, yielding an overall accuracy of 90.50%. In comparison, the MEOWS chart exhibited a sensitivity of 70.51%, specificity of 86.81%, predictive value of 92.94%, and accuracy of 83.71%.

Conclusion

The occurrence of maternal morbidity in the trigger zone was significantly higher than in the non-trigger zone in the MMS tool. The MMS tool was significantly more effective as a predictor of peripartum morbidity compared to the MEOWS chart.

## Introduction

High rates of obstetric morbidity and mortality remain a persistent problem in underdeveloped nations, even with the implementation of national and international healthcare programs [1]. By 2030, the worldwide maternal mortality rate (MMR) is expected to drop to below 70 per 100,000 live births, according to the Sustainable Development Goals (SDG) [2]. The MMR in India is currently 97 per 100,000 live births, with noticeable regional differences. Maharashtra's MMR is 33 per 100,000 live births, while Kerala boasts a unique MMR of 19 per 100,000 live births, attributed to strong referral networks and efficient screening procedures [3]. Despite continuous endeavours, India has not achieved the Millennium Development Goal's (MDG) objective of reducing maternal mortality. There is now a shift in focus to indicators such as obstetric morbidity and the ratio of severe maternal morbidity to mortality [4,5].

In India, anaemia stands as the second leading cause of maternal mortality, accounting for approximately 20% of deaths attributed to this condition [6]. A patient diagnosed with mild preeclampsia on clinical examination may have underlying multisystem involvement and related complications, primarily detected through biochemical tests [7]. The International Federation of Gynecology and Obstetrics (FIGO) also recommends that patients with preeclampsia undergo timely diagnostic biochemical testing for systemic involvement. Gestational diabetes mellitus (GDM) impacts 2%-5% of pregnancies each year. Throughout pregnancy, there is a 50% rise in the glomerular filtration rate. Nevertheless, elevated creatinine levels nearing the upper threshold of normal serve as an indicator of potential renal issues in GDM. Elevated uric acid levels observed in GDM are part of the metabolic syndrome, causing insulin resistance. The pathophysiology, biochemical, and metabolic abnormalities in GDM affect the renal system [8].

This study compares obstetric morbidity in patients using a newly developed maternal morbidity screening (MMS) tool and the modified early obstetric warning system (MEOWS) chart, assessing sensitivity, specificity, predictive value, and accuracy. To enhance predictability, we propose incorporating biochemical parameters into the screening tool format for comprehensive and accessible use by healthcare professionals. This will facilitate early identification and management of peripartum morbidity in both high- and low-risk groups.

## Materials and methods

Study setting, study design, and sample size

The present study was conducted in the Obstetrics and Gynaecology Department of Acharya Vinoba Bhave Rural Hospital (AVBRH), Sawangi (M) Wardha. The study design was an observational analytical clinical study. The study population consisted of 441 prenatal women in labour with a gestational age greater than 28 weeks. The study was approved by Datta Meghe Institute of Medical Sciences (Deemed to be University) Institutional Ethics Committee (DMIMS (DU)/IEC/2020-21/9155).

Inclusion and exclusion criteria

Eligible participants were those who were in labour and had a gestational age of more than 28 weeks. Patients who did not give consent and cases where the pregnancy continued beyond the following day were excluded.

Data collection process and instrument

The Confidential Enquiry into Maternal and Child Health (CEMACH) report's recommended MEOWS chart was used, and parameters were entered for every patient in accordance with the usual procedure [9]. The trigger zone type was derived using standard values from the MEOWS chart. Both groups' baseline measurements included heart rate, respiration rate, blood pressure, temperature, overall health, neural responsiveness, and oxygen saturation. Physiological and biochemical parameters were monitored using the MMS tool, categorizing them into trigger and non-trigger zones, as detailed in Table 1. Monitoring occurred every four hours until 24 hours post-delivery in both groups.

**Table 1:** MMS tool parameters of trigger thresholds for maternal morbidity screening Hb: haemoglobin; RBS: random blood sugar; WBC: white blood cell; SGPT: serum glutamic pyruvic transaminase; BP: blood pressure; O_2_: oxygen

Sr. No.	Parameters	Normal (Green)	Low Risk (Orange Alert)	High Risk (Red Alert)
1	Temperature	36-38 °C	35-<36 °C	>38 °C or <35 °C
2	O_2_ saturation	95-100%	-	<95%
3	Heart rate	50-100	>100-120 or 40-<50	<40 or >120
4	Respiratory rate	10-20	21-30	<10 or >30
5	Systolic BP	100-140	90-<100 or >140-160	<90 or >160
6	Diastolic BP	<90	90-100	>100
7	Proteinuria	nil-trace	1+ to 2+	>2+
9	Neural response	Alert	Responds to verbal stimuli	Unresponsive, responds to pain
10	General condition	Looks well	Looks unwell	-
11	Blood investigation Hb	11-12 g/dL	<11 g/dL	<6 g/dL
12	WBC	4.5-11.0x10^9^/L	<4.5-11.0x10^9^/L	<3.5 or irrespective
13	Platelet	150-400x10^9^/L	<150-400x10^9^/L	<10-20x10^9^/L
14	SGPT	7-56 IU/L	-	>7-56 IU/L
15	Bilirubin	1.2 mg/dL	-	>1.2 mg/dL
16	Urea	17-43 mg/dL	-	>17-43 mg/dL
17	Creatinine	0.7-1.2 mg/dL	-	>0.7-1.2 mg/dL
18	RBS	<200 mg/dL	-	>200 mg/dL

Statistical analysis

The gathered data were analyzed using both inferential and descriptive statistical techniques. This involved performing chi-square tests and examining key test statistics such as specificity, sensitivity, accuracy, and predictive value. Statistical analysis were conducted using IBM SPSS Statistics for Windows, Version 26 (Released 2019; IBM Corp., Armonk, New York), revealing significant findings with a threshold set at p<0.05.

## Results

The MMS tool identified trigger zones with a rate of 25.79%, compared to 23.07% in the MEOWS chart. The non-trigger group in the MMS observed a rate of 74.20%, while the MEOWS chart showed 76.92% (Table 2).

**Table 2:** Distribution of total patients according to the trigger and non-trigger zones on the MMS tool and MEOWS chart The percentages of patients in the trigger and non-trigger zones, as depicted by numbers MMS: maternal morbidity screening; MEOWS: modified early obstetric warning system

MMS Tool (221)		MEOWS Chart (221)	
Triggered group	Non-trigger group	Triggered group	Non-trigger group
57	164	51	170
25.79%	74.20%	23.07%	76.92%

The MMS tool and MEOWS chart showed variations in the following parameters: respiratory rate (1.35%), oxygen saturation (0.45%), temperature (0.45%), heart rate (0.45%), diastolic blood pressure (2.26%), neural response (0.90%), and general condition (0.91%). There was no significant difference found between both groups (Table 3).

**Table 3:** Comparison of the MMS group vs. the MEOWS group from the physiological parameters with the trigger zone The physiological parameters were depicted in percentages MMS: maternal morbidity screening; MEOWS: modified early obstetric warning system; BP: blood pressure

Parameters	Total Orange + Red Trigger (Abnormal Zone) MMS Tool	Total Orange + Red Trigger (Abnormal Zone) MEOWS Chart	Difference
Respiratory rate	12 (5.42%)	9 (4.07%)	1.35%
Oxygen saturation	3 (1.35%)	4 (1.80%)	0.45%
Temperature	4 (1.80%)	5 (2.26%)	0.45%
Heart rate	10 (4.52%)	11 (4.97%)	0.45%
Systolic BP	12 (5.42%)	12 (5.42%)	0
Diastolic BP	16 (7.23%)	11 (4.97%)	2.26%
Colour of liquor	2 (0.90%)	2 (0.90%)	0
Neural response	4 (1.80%)	3 (0.90%)	0.90%
General condition	3 (1.35%)	5 (2.26%)	0.91%

The most common parameters leading to derangement and placing the patient into the trigger zone were haemoglobin (Hb) (5.88%), proteinuria (6.78%), serum glutamic pyruvic transaminase (SGPT) (4.07%), white blood cell (WBC) (4.07%), and random blood sugar (RBS) (3.16%) (Table 4).

**Table 4:** The correlation between the individual maternal morbidity screening (MMS) tool and the trigger zones based on biochemical parameters The biochemical parameters were depicted in percentages Hb: haemoglobin; RBS: random blood sugar; WBC: white blood cell; SGPT: serum glutamic pyruvic transaminase

Parameters	Green Zone (Normal Zone)	Orange Zone	Red Zone	Total Orange + Red Trigger (Abnormal Zone)
Hb	208	10	3	13 (5.88%)
RBS	214	5	2	7 (3.16%)
WBC	212	6	3	9 (4.07%)
Platelets	218	2	1	3 (1.35%)
SGPT	212	9	-	9 (4.07%)
Bilirubin	219	-	2	2 (0.90%)
Proteinuria	206	7	8	15 (6.78%)
Creatinine	213	5	3	8 (3.61%)
Urea	217	3	1	4 (1.80%)

The analysis demonstrated significant improvement in morbidity during hospital stay, 66.66% in the MMS tool and 32% in the MEOWS chart. The MMS tool had more likelihood of developing morbidity than the MEOWS chart (Table 5).

**Table 5:** Comparison of maternal morbidity during hospital stays among abnormal and normal clients with the MMS tool and MEOWS chart Category 1: The patient did not have any obstetric morbidity during hospital stay Category 2: The patient had morbidity during hospital stay The data have been presented in percentages MMS: maternal morbidity screening; MEOWS: modified early obstetric warning system

	MMS Tool		MEOWS Chart	
Obstetric morbidity during hospital stay	Triggered group (n=57)	Non-triggered group (n=164)	Triggered group (n=51)	Non-triggered group (n=170)
Category 1	19 (33.33%)	162 (98.78%)	27 (68%)	158 (88.77%)
Category 2	38 (66.66%)	2 (1.21%)	24 (32%)	12 (11.22%)
Total	57 (100%)	164 (100%)	51 (100%)	170 (100%)

The normal delivery rate in the MMS tool for the triggered group is 61.40%, while the rate for the lower-segment caesarean section (LSCS) is 30.08 % (Table 6).

**Table 6:** Comparison of the delivery methods and obstetric interventions in the normal (trigger and non-trigger) groups p-value<0.05 is considered significant LSCS: lower-segment caesarean section; x^2^: chi-square

Mode of Delivery and Obstetric Intervention	Normal (Triggered) Group (n=57)	Abnormal (Non-Triggered) Group (n=164)	χ2-value
Normal delivery	35 (61.40%)	54 (32.92%)	16.65, p=0.0002, S
LSCS	20 (30.08%)	108 (65.85%)	
Assisted	2 (3.50)	2 (3.50%)	
Total	57 (100%)	164 (100%)	

The sensitivity of the MMS tool was found to be 95.24%, compared to 70.51% for the MEOWS chart. The specificity of the MMS tool was 89.50% compared to 86.81% for the MEOWS. The predictive value of the MMS was 98.50% compared to 92.94% for the MEOWS, and the accuracy of the MMS was 90.50% as opposed to 83.71% for the MEOWS chart. The MMS tool had more sensitivity, specificity, predictive value, and accuracy than the MEOWS chart (Table 7).

**Table 7:** Assessing the effectiveness of the maternal morbidity screening (MMS) tool with MEOWS chart Performance metrics for the MMS group and MEOWS chart in detecting maternal morbidity, facilitating comparison of their effectiveness in terms of sensitivity, specificity, predictive value, and accuracy. This identifies those with and without maternal morbidity and the accuracy of positive and negative test results, presented as percentages MMS: maternal morbidity screening; MEOWS: modified early obstetric warning system

Maternal Morbidity	MMS Tool	MEOWS Chart
Sensitivity	95.24%	70.51%
Specificity	89.50%	86.81%
Positive predictive value	70.24%	52.95%
Negative predictive value	98.50%	92.94%
Accuracy	90.50%	83.71%

## Discussion

In the MMS tool, the trigger zone was 25.79%, compared to 23.07% in the MEOWS chart. The non-trigger zone in the MMS group was 74.20%, while in the MEOWS chart, it was 76.92%. This comparison aligns with the studies conducted by Singh A et al. [10] and Singh S et al. [11]. In these studies, among the triggered group, 66.66% fell into category II, indicating those with obstetric morbidity in the MMS tool and 32% in the MEOWS chart. The MMS tool showed a higher likelihood of developing morbidity compared to the MEOWS chart. Similar results were observed in the study conducted by Singhal S et al. [12]. In the non-triggered group, 1.21% were categorized under category II, with the most prevalent obstetric morbidity being hypertensive disorders of pregnancy at 1.21%, followed by anaemia at 1.21% and gestational diabetes at 0.60%. Consequently, it can be deduced that the most common obstetric morbidities in both the category I and category II groups were hypertensive disorders of pregnancy and anaemia.

Research conducted by Singh et al. reported obstetric morbidity in 26.6% of the triggered group and 16.61% of the non-triggered group. The study noted one mortality in the triggered group, attributed to eclampsia. Hypertensive disorders of pregnancy emerged as the most prevalent morbidity in the triggered group, followed by anaemia, obstetric haemorrhage, and sepsis [10]. The findings were comparable with the current study. In a study by Singh S et al., there were no reported cases of maternal mortality [11]. However, in contrast to the present study, haemorrhage emerged as the predominant morbidity, followed by hypertensive disorders in pregnancy and anaemia. A study conducted in developing countries by Khan KS et al. examined the collective factors contributing to maternal deaths using various datasets. It was concluded that haemorrhage and hypertensive disorders were the primary factors leading to maternal deaths in developing nations [13].

The study conducted by Geller SE et al. assessed factors associated with maternal outcomes, revealing haemorrhage and hypertensive disorders as the principal causes, although with some regional variations [14]. In the current study, hypertensive disorders of pregnancy stand out as the predominant cause of obstetric morbidity. One possible reason for this could be the low awareness levels among patients. Anaemia, being the second most common cause of morbidity in triggered patients in the present study, might reflect a lack of proper iron supplementation, poor nutritional status, and the occurrence of multiple and more frequent births among Indian females. Therefore, addressing this issue requires intensive care and efforts to improve maternal outcomes significantly.

Haemoglobin levels found at 5.88%, which is considered abnormal, are a crucial biochemical parameter in pregnancy. Early recognition of low haemoglobin can prevent further complications during pregnancy. Several studies have reported it to be one of the most common issues encountered [15,16]. There was an elevation in proteinuria by 6.78%, with 5.42% and 4.97% increases in systolic blood pressure (SBP) and diastolic blood pressure (DBP), respectively. Both SBP and DBP were significantly higher in preeclampsia patients. Several studies have also reported these findings [17,18]. Routine measurement of blood pressure during pregnancy has long been a standard part of prenatal care [19].

The sensitivity of the MMS screening tool was 95.24%, the specificity was 89.50 %, and the predictive value was 98.50%. The accuracy of the MMS tool was 90.50%. The MEOWS chart showed a sensitivity of 70.51%, specificity of 86.81%, predictive value of 92.94%, and accuracy of 83.71%. It indicates that the MMS tool was more efficacious for predicting peripartum morbidity. The sensitivity in the MMS tool is higher than in the previous research by Singhal S et al. [12,20]. Healthcare workers should consider researching common disease entities leading to obstetric morbidity at healthcare systems' primary, secondary, and tertiary levels.

Clinical implications

The MMS tool is a potentially valuable and cost-effective resource, providing a straightforward means for healthcare workers to promptly identify, treat, and urgently refer cases.

Strengths

This study categorizes the trigger and non-trigger zones and compares them with the MEOWS chart. The MMS tool includes both physiological and biochemical parameters, enabling the early detection of peripartum morbidity before it reaches severe levels.

Limitations

As such, there are no palpable weaknesses; however, long-term follow-up is required to assess.

## Conclusions

The occurrence of maternal morbidity in the trigger zone was significantly higher than in the non-trigger zone in the MMS tool compared with the MEOWS chart. The MMS tool demonstrated superior predictive capability for peripartum morbidity compared to the existing chart. Overall, the accuracy of the MMS tool in predicting peripartum morbidity was significantly higher than that of the MEOWS chart. Consequently, employing the MMS screening tool to anticipate peripartum morbidity is recommended.
